# Correction of humpback and DISI deformities by vascularized bone grafting in patients with scaphoid nonunion

**DOI:** 10.1051/sicotj/2021011

**Published:** 2021-03-11

**Authors:** Nana Nagura, Kiyohito Naito, Yoichi Sugiyama, Hiroyuki Obata, Kenji Goto, Ayaka Kaneko, Yoshimasa Tomita, Yoshiyuki Iwase, Kazuo Kaneko, Muneaki Ishijima

**Affiliations:** 1 Department of Orthopaedics, Juntendo University Faculty of Medicine 2-1-1 Hongo Bunkyo-ku 113-8421 Tokyo Japan; 2 Department of Medicine for Orthopaedics and Motor Organ, Juntendo University Graduate School of Medicine 2-1-1 Hongo Bunkyo-ku 113-8421 Tokyo Japan; 3 Department of Orthopeadic Surgery, Juntendo Tokyo Koto Geriatric Medical Center 3-3-20 Shisuna Koto-ku 136-0075 Tokyo Japan; 4 Department of Orthopaedic Surgery, Japan Labour Health and Safety Organization Tokyo Rosai Hospital 4-13-21 Omori-Minami Ota-ku 143-0013 Tokyo Japan

**Keywords:** Scaphoid nonunion, Humpback deformity, Dorsal intercalated segment instability deformity, The time to surgery, 1, 2 intercompartmental supraretinacular artery

## Abstract

*Introduction*: Although vascularized bone grafting (VBG) using 1, 2 intercompartmental supraretinacular artery (1, 2 ICSRA) is effective for scaphoid nonunion, dorsal intercalated segment instability (DISI) deformity persists even after correction of humpback deformity (HD). The purpose of this retrospective study was to evaluate the correction of HD and DISI deformity after 1, 2 ICSRA VBG for scaphoid nonunion. *Methods*: We treated 18 patients (mean age: 25.8, 16 males and 2 females) with scaphoid nonunion using a 1, 2-ICSRA VBG between January 2010 and December 2018. The average time from injury to surgery was 20.0 (3–120) months. The nonunions were located at the waist in all patients. The correction of HD and DISI deformity was investigated on the preoperative images and images at the last examination. *Results*: In all patients, the correction of HD was positively correlated with that of DISI deformity. Moreover, we focused on the time from injury to surgery and evaluated changes in HD and DISI deformity according to the time to surgery. As a result, changes in HD and DISI deformity were positively correlated in patients with a shorter time to surgery but were not correlated when the time to surgery exceeded 5 months. *Conclusions*: These results suggest that DISI deformity can be corrected by correcting HD when the time from injury to surgery is short, but that correction is difficult if the time to surgery is prolonged.

## Introduction

Vascularized pedicled radial grafting for scaphoid nonunion reported in 1991 by Zaidemberg (Z procedure) is autologous bone grafting using the ascending irrigating branch of the radius, i.e., 1, 2 intercompartmental supraretinacular artery (1, 2 ICSRA) as the feeding vessel [1]. Vascularized bone grafting (VBG) is effective for the treatment of scaphoid nonunion, particularly, in refractory cases, such as those accompanied by avascular necrosis (AVN), those with a history of surgery, those with a long period after injury, and those of the proximal third of the scaphoid [2]. The outcomes of the Z procedure are satisfactory in refractory cases [3, 4].

On the other hand, Ribak et al. compared the outcomes of VBG and non-VBG for scaphoid nonunion including nonunion in the waist. As a result, it was reported that VBG had a higher bone union rate and a shorter time to a bone union than non-VBG [5]. From this evidence, we use VBG to treat scaphoid nonunions in cases of poor blood flow in proximal bone fragments and cases in which the time from injury to surgery is long, even for nonunions in the waist.

One of the factors for dorsal intercalated segment instability (DISI) deformity associated with scaphoid nonunion is humpback deformity (HD) due to bone defect on the volar side of the fracture site [6]. Moreover, the proximal fragment flexes dorsally with the lunate bone to cause DISI deformity [7, 8], and persistence of DISI deformity is reported to be a factor of poor clinical prognosis [9, 10]. Therefore, correction of HD and DISI deformity is important along with bone union for improving the clinical outcome of nonunion and preventing scaphoid nonunion advanced collapse (SNAC) wrist. However, the surgical technique in the treatment of scaphoid nonunion is still controversial. Kim et al. reported autologous iliac bone grafts for scaphoid nonunion showed a significant correlation between correction of HD and correction of DISI deformity [9]. On the other hand, in the Z procedure, where the amount of bone graft is limited, the reduction that can be obtained after surgery remains questionable [11]. Moreover, there is no literature describing the correlation between correction of both deformities after VBG for scaphoid nonunion. Therefore, the correction of HD and DISI deformity after VBG for scaphoid nonunion were evaluated, then the relationship between the degree of correction of HD and DISI deformity and the time to surgery was evaluated in this study.

## Materials and methods

### Patients

This study was approved by the ethics committee for medical research of our university (No. 18-323), and informed consent was received from all patients and the patients of minor patients under the age of 20.

Between January 2010 and December 2018, 1, 2 ICSRA VBG using was performed for scaphoid nonunion in 18 patients with a mean age of 25.8 (16–46) years consisting of 16 males and 2 females. The average time from injury to surgery was 20.0 (3–120) months. The fracture type at the time of injury by the Herbert classification was B1 in 6 and B2 in 12, and the nonunions were located at the waist in all patients.

### Surgical technique

Surgery was performed under general anesthesia. According to the report by Zaidemberg et al., the 1, 2 ICSRA running dorsally between the first and second compartments of the extensor tendon was identified, a graft including this vessel was raised from the radius and placed at the site of nonunion [1]. Curettage of the site of nonunion was performed from the volar side in patients with relatively large HD but from the dorsal side in the others, and the size of the graft to be placed was measured. In patients judged by preoperative imaging to have relatively severe DISI deformity, the radius and lunate bone were temporarily fixed for intraoperative correction of DISI deformity by inserting a Kirschner wire from the dorsal side of the radius toward the lunate bone while correcting the radiolunate (RL) angle to 0° by volarly flexing the wrist, and the graft was placed in this condition. Headless compression screws were used for the fixation of the nonunion. After surgery, thumb spica splinting was applied for 4 weeks, and range of motion exercise was initiated thereafter.

### Imaging assessment

To evaluate the degree of correction of HD and DISI deformity by 1, 2 ICSRA VBG for scaphoid nonunion, the preoperative images and images at the last examination by plain radiography and computed tomography (CT) were compared. At our hospital, preoperative CT was used to evaluate the condition of the nonunion site and scaphoid alignment, and postoperative CT was used to evaluate bone union and scaphoid alignment in all patients. The intrascaphoid angle (ISA) was examined as an index of HD, and the scapholunate angle (SLA) was examined as an index of DISI deformity. ISA is an angle formed by two lines that are perpendicular to the proximal and distal articular surfaces in the lateral view on CT (Figure 1A), and SLA is the angle between the long axis of the scaphoid (tangent line of the volar cortex of the scaphoid) and the long axis of the lunate bone (the line between the midpoints of the proximal and distal arcs) in the lateral view of plain radiography of the wrist (Figure 1B). Measurements of ISA and SLA were performed by an independent blind orthopaedic surgeon.

**Figure 1:**
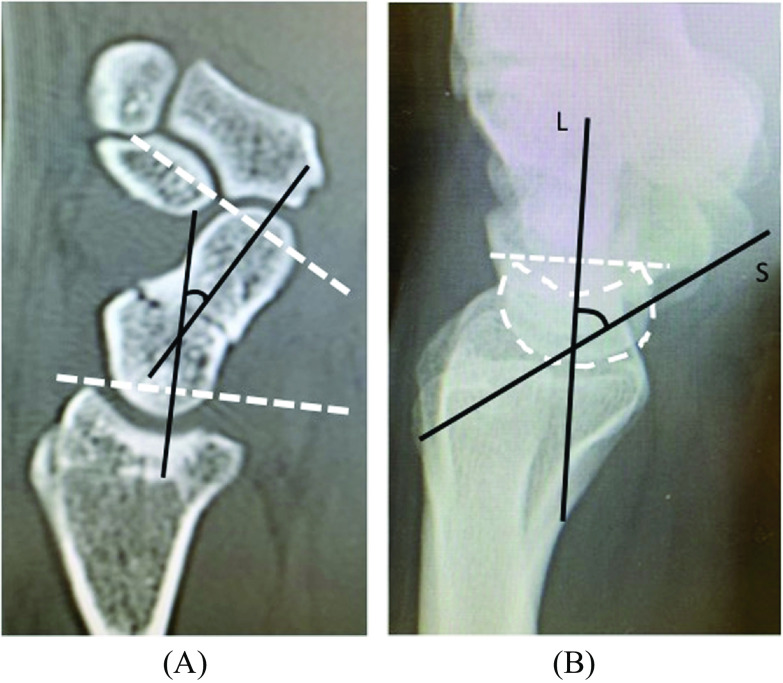
Method for the measurement of the intrascaphoid angle (ISA) (A) and scapholunate angle (SLA) (B). (A) ISA was measured as the angle between the perpendicular lines on the distal and proximal articular surfaces of the scaphoid in the lateral CT image. (B) SLA was measured as the angle between the long axis of the scaphoid (tangent on the volar cortex of the scaphoid) and the long axis of the lunate (axis connecting the midpoints of the proximal and distal arcs) in the lateral view of plain radiography of the wrist.

### Evaluations

In this study, to assess the improvement in HD and DISI deformity, ISA and SLA were compared preoperatively and at the time of the last postoperative examination. In addition, the relationship between the degree of correction of HD and DISI deformity and the time to surgery was evaluated according to the time after injury (<4 months vs. ≥4 months, <5 months vs. ≥5 months, and <6 months vs. ≥6 months).

### Statistical analysis

The data obtained by imaging examinations were compared using the Mann–Whitney *U*-test. The correlation of the changes in the data between before and after surgery was evaluated by calculating Spearman’s correlation coefficient. The level of significance was *p* < 0.05.

## Results

### Measurements of ISA and SLA, and the correlation between ISA and SLA in all patients

The mean postoperative follow-up period was 15.7 ± 2.5 months. Of the 18 patients, nonunion was healed in 15 and not in 3, with a union rate of 83.3%. The mean time to union was 4.0 (2–7) months. ISA determined by CT was 37.8 ± 18.5° preoperatively and was significantly improved to 20.2 ± 12.9° at the time of last postoperative examination (*p* < 0.05). SLA determined by plain radiography was 63.9 ± 12.7° preoperatively and improved to 52.6 ± 18.6° at the time of the last examination (*p* = 0.06) (Table 1). The changes in ISA and SLA at the time of the last examination compared with the preoperative values were 17.5 ± 14.9 and 8.9 ± 11.2, respectively, and showed a positive correlation (*r* = 0.54, *p* < 0.05) (Table 2).

**Table 1:** Period from injury to surgery and changes in the intrascaphoid angle (ISA) and scapholunate angle (SLA) between before and after surgery.

	ISA			SLA		
	Before	After	Change	Before	After	Change
All patients (*n* = 18)	37.8 ± 18.5	20.2* ± 12.9	17.5 ± 14.9	63.9 ± 12.7	52.6 ± 18.6	8.9 ± 11.2
Time to surgery < 4 months (*n* = 5)	36.4 ± 22.6	18.6 ± 11.5	17.8 ± 16.0	60.2 ± 15.3	52.6 ± 13.3	7.6 ± 11.8
Time to surgery ≥ 4 months (*n* = 13)	38.4 ± 17.7	20.9* ± 13.8	17.5 ± 15.1	65.3 ± 11.9	52.7 ± 20.7	9.4 ± 11.4
Time to surgery < 5 months (*n* = 9)	41.9 ± 18.3	23.0* ± 14.1	18.9 ± 17.5	61.6 ± 13.4	56.0 ± 14.4	6.9 ± 11.5
Time to surgery ≥ 5 months (*n* = 9)	33.7 ± 18.9	17.6* ± 11.7	16.2 ± 12.7	66.2 ± 12.3	49.6 ± 22.1	10.9 ± 11.2
Time to surgery < 6 months (*n* = 11)	41.9 ± 19.1	20.5* ± 14.5	21.4 ± 16.8	65.2 ± 15.0	55.9 ± 14.2	10.5 ± 12.4
Time to surgery ≥ 6 months (*n* = 7)	34.3 ± 18.4	19.7 ± 12.4	14.6 ± 10.7	63.7 ± 9.7	55.6 ± 16.0	7.9 ± 10.3

Statistical analysis of the difference between before and after surgery: *p < 0.05.

ISA: Intrascaphoid angle, SLA: Scapholunate angle.

**Table 2:** Correlations between the intrascaphoid angle (ISA) and scapholunate angle (SLA).

All patients (*n* = 18)	
0.54*	
Time to surgery < 4 months (*n* = 5)	Time to surgery ≥ 4 months (*n* = 13)
*r* = 0.78*	*r* = 0.42*
Time to surgery < 5 months (*n* = 9)	Time to surgery ≥ 5 months (*n* = 9)
*r* = 0.89*	*r* = 0.18
Time to surgery < 6 months (*n* = 11)	Time to surgery ≥ 6 months (*n* = 7)
*r* = 0.79*	*r* = −0.11

Statistical analysis of the correlation between ISA and SLA: *p < 0.05 and |*r*_*s*_| > 0.4.

ISA: Intrascaphoid angle, SLA: Scapholunate angle.

### Comparison according to the time to surgery (<4 months vs. ≥4 months)

In the 5 patients (5 males and 0 females) in whom the time to surgery was < 4 months, ISA was 36.4 ± 22.6° preoperatively and improved to 18.6 ± 11.5° at the time of the last examination (*p* = 0.13), and SLA improved from 60.2 ± 15.3° before surgery to 52.6 ± 13.3° at the time of the last examination (*p* = 0.33) (Table 1). In the 13 patients (11 males and 2 females) in whom the time to surgery was ≥ 4 months, ISA significantly improved from 38.4 ± 17.7° before surgery to 20.9 ± 13.8° at the time of the last examination (*p* < 0.05), and SLA improved from 65.3 ± 11.9° before surgery to 52.7 ± 20.7° at the time of the last examination (*p* = 0.09). The change in ISA from before surgery to the time at the last examination was 17.8 ± 16.0 in the <4 months group and 17.5 ± 15.1 in the ≥4 months group and the change in SLA was 7.6 ± 11.8 and 9.4 ± 11.4, respectively. The changes in ISA and SLA were correlated in both groups (*r* = 0.78, *p* < 0.05; *r* = 0.42, *p* < 0.05) (Table 2).

### Comparison according to the time to surgery (<5 months vs. ≥5 months)

In the 9 patients (9 males and 0 females) in whom the time to surgery was < 5 months, ISA significantly improved from 41.9 ± 18.3° before surgery to 23.0 ± 14.1° at the time of the last examination (*p* < 0.05), and SLA improved from 61.6 ± 13.4° to 56.0 ± 14.4° (*p* = 0.31) (Table 1). In the 9 patients in whom the time to surgery was ≥ 5 months (7 males and 2 females), ISA improved significantly from 33.7 ± 18.9° before surgery to 17.6 ± 11.7° at the time of the last examination (*p* < 0.05), and SLA improved from 66.2 ± 12.3° to 49.6 ± 22.1° (*p* = 0.11). The change in ISA from before surgery to the time of the last examination was 18.9 ± 17.5 in the <5 months group and 16.2 ± 12.7 in the ≥5 months group and the change in SLA was 6.9 ± 11.5 and 10.9 ± 11.2, respectively. The changes in ISA and SLA were positively correlated (*r* = 0.89, *p* < 0.05) in the <5 months group but not in the ≥5 months group (*r* = 0.18, *p* > 0.05) (Table 2).

### Comparison according to the time to surgery (<6 months vs. ≥6 months)

In the 11 patients (10 males and 1 female) in whom the time to surgery was < 6 months, ISA improved from 41.9 ± 19.1° before surgery to 20.5 ± 14.5° at the time of the last examination (*p* = 0.06), and SLA improved from 65.2 ± 15.0° to 55.9 ± 14.2° (*p* = 0.13) (Table 1). In the 7 patients (6 males and 1 female) in whom the time to surgery was ≥ 6 months, ISA significantly improved from 34.3 ± 18.4° before surgery to 19.7 ± 12.4° at the time of the last examination (*p* = 0.06), and SLA improved from 63.7 ± 9.7° to 55.6 ± 16.0° (*p* = 0.27). The change in ISA from before surgery to the time of the last examination was 21.4 ± 16.8 in the <6 months group and 14.6 ± 10.7 in the ≥6 months group and the change in SLA was 10.5 ± 12.4 and 7.9 ± 10.3, respectively. The changes in ISA and SLA were positively correlated in the <6 months group (*r* = 0.79, *p* < 0.05) but not correlated in the ≥6 months group (*r* = −0.11, *p* > 0.05) (Table 2).

## Discussion

The size of bone grafts in the treatment of scaphoid nonunion is still controversial. Generally, to correct HD associated with scaphoid nonunion, it is necessary to graft a sufficient amount of bone as a support on the volar side [11]. Among the VBGs, the one placed from the volar side of the radius proposed by Judet et al. has been reported to have allowed the collection of larger grafts and resulted in a satisfactory bone union rate compared with the Z procedure, by which grafts are collected from the dorsal side of the radius [12, 13]. In addition, free VBG from the medial condyle of the femur allows the collection of larger grafts compared with other pedicled VBGs, and favorable clinical results have been reported [14]. The Z procedure is inferior to these techniques in the graft size [11, 15]. However, as observed above, for the treatment of refractory scaphoid nonunion, the outcomes of the Z procedure have been reported to be generally favorable, and the technique is a useful option of surgical treatment [3, 4, 13].

There are several limitations in this study. The first is a method for evaluation of the alignment of the scaphoid. Recently, several studies using the scaphoid length as an indicator of reduction and carpal alignment were reported [16, 17]. Moreover, Kim et al. reported that the shortening of the scaphoid caused DISI deformity [9]. However, the scaphoid length has a gender difference and laterality [18, 19], and it can be difficult to use it as an evaluation for the reduction of the scaphoid nonunion. Therefore, in the cases of this study, when the Z procedure was performed from the volar side, the continuity of the dorsal site was maintained, and bone grafting was performed on the wedge-shaped bone defect to reduce the alignment. In addition, when the Z procedure was performed from the dorsal side, the continuity of the volar site was also maintained. Based on these findings, although the shortening of the scaphoid itself has not been strictly evaluated, ISA was used as the evaluation of HD in this study. Therefore, it cannot be asserted that the cause of residual DISI deformity in this study is only the involvement of subclinical damage to the soft tissues such as the SL ligament. Next, the indication of the Z procedure is still controversial. In previous reports, Chang et al. reported that the Z procedure was performed on nonunions with HD and AVN, but the bone union was not obtained and good treatment results could not be obtained [15]. In addition, Derby et al. reported that VBG was relatively contraindicated in cases with HD and carpal instability [20]. However, in Japan, Kawasaki et al. reported favorable outcomes of the Z procedure and the correction of DISI for scaphoid nonunion with HD [21]. And there is a current situation among Japanese hand surgeons that, contrary to reports in previous English literature, the Z procedure is preferred for this nonunion. In fact, we have also found improvement in HD using the Z procedure for cases with HD, and we think that the Z procedure may be useful for cases with a relatively short time to injury.

Indeed, in our patients, ISA improved significantly at the time of the last examination compared with before surgery, indicating that the Z procedure allows the collection of a sufficient amount of bone for correction of HD (Table 1). In addition, the change in ISA at the time of the last examination compared with the value before surgery was positively correlated with the change in SLA, indicating that DISI deformity was improved by correcting HD (Table 2). However, in some of the 18 patients evaluated in this study, correction of DISI deformity was insufficient even when HD could be corrected.

Concerning correction of DISI associated with scaphoid nonunion, Fisk et al. reported in a study about wedge grafts that the volar radiocarpal ligament tension could be normalized, and DISI could be corrected, by the restoration of the length of the scaphoid bone shortened by HD [6]. Although the SL ligament is the primary ligament that contributes to scaphoid stability, the radioscaphocapitate (RSC) ligament and long RL ligament, which are volar radiocarpal ligaments, are reported to contribute secondarily to stability [22]. Therefore, we directed our attention to the ligaments around the scaphoid bone as a factor of insufficient correction of DISI despite correction of HD. We hypothesized that the history of HD is prolonged in long-standing scaphoid nonunion and that correction of DISI deformity is made difficult by changes in the length and elasticity of the surrounding ligaments despite the possibility of bony correction of HD. In this study, we focused on the period from injury to surgery and evaluated changes in HD and DISI deformity according to the time to surgery (<4, 5, and 6 months vs. ≥4, 5, and 6 months). As a result, changes in HD and DISI deformity were positively correlated in patients with a shorter time to surgery but were not correlated when the time to surgery exceeded 5 months (Table 2). These results suggest that DISI deformity can be corrected by correcting HD when the time from injury to surgery is short but that correction of DISI deformity by correction of HD is difficult if the time to surgery is prolonged. Thus, in patients with scaphoid nonunion, HD may be corrected, but DISI may not be sufficiently corrected, by the Z procedure if the time from injury to surgery is long.

Capito and Higgins prepared models mimicking scaphoid nonunion accompanied by DISI deformity using cadavers by cutting the SL ligament, which stabilizes the scaphoid bone, RSC ligament, and long RL ligament [22]. They showed that DISI deformity can be corrected without restoration of the ligaments by inserting a bone graft slightly larger than the actual bone defect [22]. Our present study suggested that the Z procedure is a useful option for surgical treatment of scaphoid nonunion but that its effectiveness for the correction of DISI deformity is questionable if surgery is performed more than 5 months after injury, and the effect of intracarpal ligaments is considered a factor of such an outcome.

## Declarations

### Funding

The authors declare that no funding was involved in this study.

### Conflict of interest

The authors declare that they have no conflict of interest.

### Ethics approval

The study was approved by the ethics committee for medical research of our university (No. 18-323).

### Consent to participate

Informed consent was received from all patients.

### Consent for publication

Informed consent was received from all patients.

### Availability of data and material

The datasets during and/or analysed during the current study available from the corresponding author on reasonable request.

### Code availability

The datasets during and/or analysed during the current study available from the corresponding author on reasonable request.

### Authors’ contributions

NN (first author) mainly wrote this manuscript, acquisition of data, analysis and interpretation of data. KN (corresponding author), YS, YI, KK and MI mainly performed conception and design of this study. HO, KG, AK, and YT performed acquisition of data, analysis and interpretation of data.
